# The local and global climate forcings induced inhomogeneity of Indian rainfall

**DOI:** 10.1038/s41598-018-24021-x

**Published:** 2018-04-16

**Authors:** P. J. Nair, A. Chakraborty, H. Varikoden, P. A. Francis, J. Kuttippurath

**Affiliations:** 1 CORAL, Indian Institute of Technology Kharagpur, West Bengal, India; 2 Indian Institute of Tropical Meteorology, Pashan, Pune 411008 India; 3 ESSO-Indian National Centre for Ocean Information Services, Hyderabad, India

## Abstract

India is home for more than a billion people and its economy is largely based on agrarian society. Therefore, rainfall received not only decides its livelihood, but also influences its water security and economy. This situation warrants continuous surveillance and analysis of Indian rainfall. These kinds of studies would also help forecasters to better tune their models for accurate weather prediction. Here, we introduce a new method for estimating variability and trends in rainfall over different climate regions of India. The method based on multiple linear regression helps to assess contributions of different remote and local climate forcings to seasonal and regional inhomogeneity in rainfall. We show that the Indian Summer Monsoon Rainfall (ISMR) variability is governed by Eastern and Central Pacific El Niño Southern Oscillation, equatorial zonal winds, Atlantic zonal mode and surface temperatures of the Arabian Sea and Bay of Bengal, and the North East Monsoon Rainfall variability is controlled by the sea surface temperature of the North Atlantic and extratropial oceans. Also, our analyses reveal significant positive trends (0.43 mm/day/dec) in the North West for ISMR in the 1979–2017 period. This study cautions against the significant changes in Indian rainfall in a perspective of global climate change.

## Introduction

Climate change has a great impact on rainfall patterns in regional and global scale. The impact of climate change on rainfall can be studied from its long-term evolution. In India, annual rainfall is largely accounted from the southwest monsoon rainfall (also termed as Indian summer monsoon rainfall – ISMR) and northeast monsoon rainfall (NEMR). The ISMR is characterized by the flow of winds from the southwest to the land that brings moisture from the Indian Ocean to the Indian subcontinent during the summer months of June, July, August and September. The rainfall in these months is very important for cultivation. The NEMR is driven by the winds blown from the Indian subcontinent to the Indian Ocean in the northeast direction during the winter months of October, November and December. Indian rainfall is a highly variable, complex system that depends on several atmospheric and oceanic processes. A very few climate forcings are known to describe its large spatio-temporal variability to date. The important teleconnection patterns known to influence the variability of Indian rainfall are sea surface temperature (SST) variations in the eastern-to-central equatorial Pacific, termed as canonical El Niño Southern Oscillation [ENSO] or eastern Pacific (EP) ENSO^1^, North Atlantic SST variability or Atlantic Multidecadal Oscillation (AMO)^2,3^, and southern equatorial SST variability or the Indian Ocean Dipole (IOD)^4^. The equatorial Indian Ocean Oscillation (EQUINOO), the atmospheric counterpart of IOD mode^5^, also plays a key role in modulating the ISMR^6^. Recently, other climate modes such as dateline ENSO or central Pacific (CP) ENSO^7^, Atlantic Zonal Mode (AZM)^8,9^ and Extratropical SST (ESST)^10^ in connection with the SST variations in the CP, Atlantic and extratropical Oceans are also explored in relation to the ISMR. Extreme weather and climate events can be explained using some of these proxies and hence, are used for forecasting rainfall. Although weather forecast is efficiently carried out in India, its accuracy is limited due to lack of adequate information on climate processes that could precisely describe the variability and inhomogeneity of Indian rainfall^11^. Therefore, accurate knowledge on these climate processes is necessary for a better analysis and prediction of rainfall in the region concerned. The previous studies have already shown correlation between ISMR and climate processes such as ENSO, IOD or EQUINOO. However, simultaneous contribution of various climate processes (either local or global) is not assessed yet, and hence, this study examines the contributions of such climate drivers on Indian rainfall across the seasons.

Several studies have been performed out on the long-term trends in Indian rainfall, and these studies find it difficult to treat India as a single grid for interpreting trends in regional rainfall, as the long-term trends have large seasonal and regional differences. For instance^12^, found significant negative trends in ISMR in Jharkhand, Chattisgarh and Kerala, and significant positive trends in other subdivisions for the 1901–2003 period. Similarly, a study over Kerala by Krishnakumar *et al*.^13^ showed insignificant positive trends during the winter and summer seasons, whereas significant negative trends in June–July months during the 1871–2005 period. Most of these analyses used Mann-Kendall statistics or Sen’s slope estimator for computing temporal evolution of rainfall. Also, the methods that use linear regression do not account for the dynamical contributions or a time-variant parameter. Therefore, we introduce a new method, multiple linear regression (MLR), to account for the aforementioned climate forcings in analysing variability and estimating trends of Indian rainfall across the seasons as this method was never applied for these kinds of studies although it was used for predicting ISMR^14–17^.

**Figure 1:**
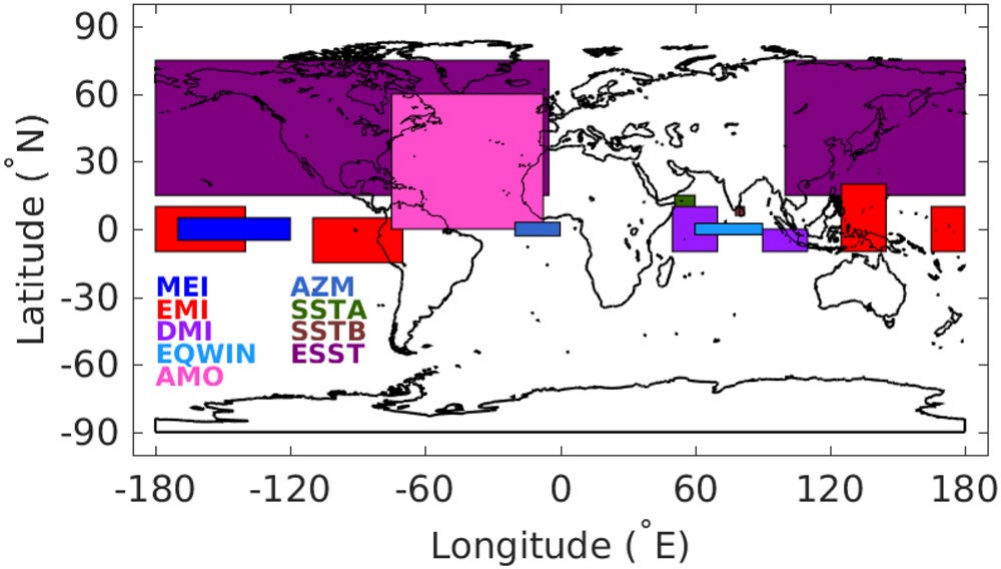
Schematic of the world map with regions considered for the climate modes are displayed in different colours as shown by the legend. The figure is created using Matlab R2016a (https://www.mathworks.com/products/new_products/release2016a.html).

Therefore, objective of this study is to (i) introduce the MLR approach to assess variability and estimate trends in Indian rainfall using various measurements for all seasons, (ii) examine simultaneous role of local and global climate processes on the interannual and seasonal variability of Indian rainfall and (iii) to explain changes in rainfall in connection with different climate forcings. The climate indices concerned are Multivariate ENSO Index (MEI), El Niño Modoki Index (EMI), AMO Index, Dipole Mode Index (DMI), Equatorial zonal Wind Index (EQWIN), AZM index, ESST index, and SST indices for the Arabian Sea (SSTA) and Bay of Bengal (SSTB) corresponding to the climate modes of EP ENSO, CP ENSO, AMO, IOD, EQUINOO, Atlantic Niño, and SST changes in the extratropial oceans, Arabian Sea and Bay of Bengal, respectively. Both MEI and EMI are used in the regression as their generation mechanisms and impact on Indian rainfall are different. Similarly, DMI and EQWIN are different sides of IOD mode, and are not significantly correlated and hence both are used. The latitude and longitude ranges considered for these climate processes are mentioned in’Methods’ and are displayed in Fig. 1.

**Figure 2:**
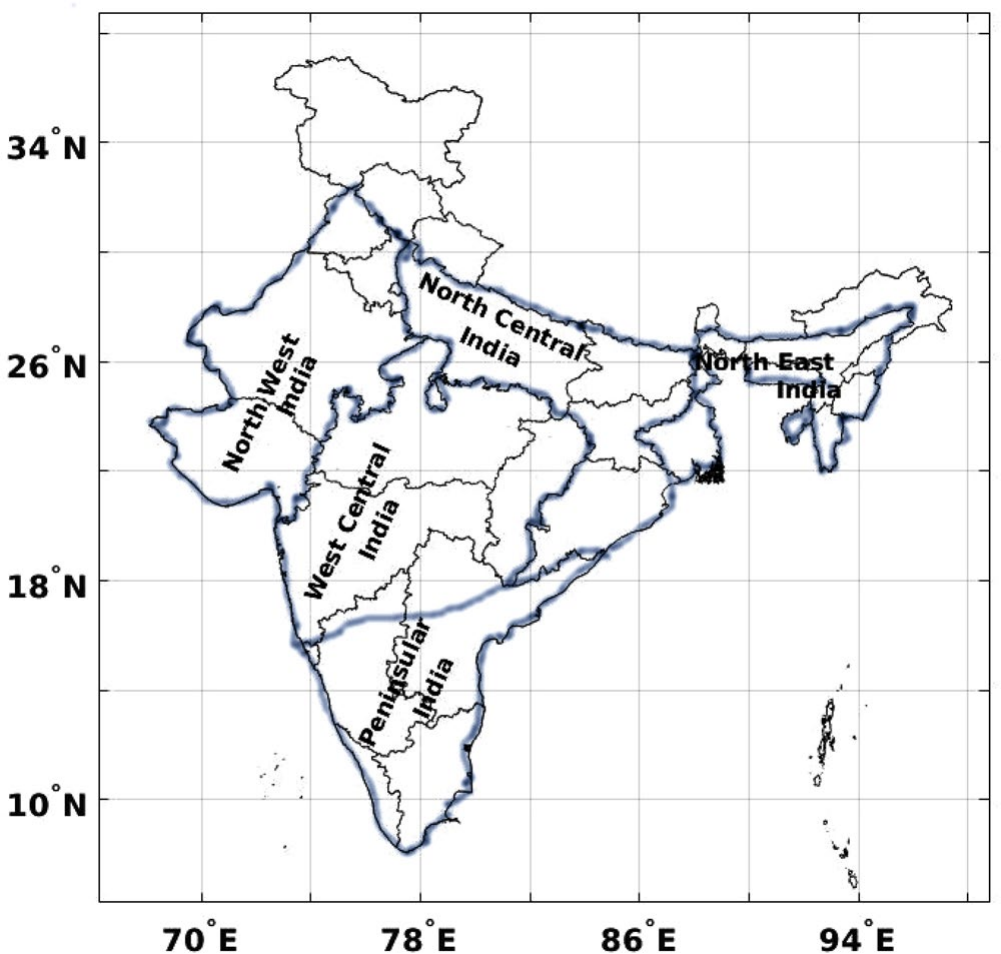
Schematic of Indian climate regions as classified by India Meteorological Department (IMD). These climate regions are considered for analysing rainfall variability and trends. The figure is created using Matlab R2016a (https://www.mathworks.com/products/new_products/release2016a.html) and modified using GNU Image Manipulation Program (GIMP) editor version 2.8.10. (http://www.gnu.org/licenses/).

The gridded rainfall data available from the India Meteorological Department (hereafter IMD data)^18^, and Global Precipitation Climatology Project (GPCP)^19^, Climate Prediction Center (CPC) Merged Analysis of Precipitation (CMAP)^20^ and Tropical Rainfall Measuring Mission (TRMM) instruments are used for the analysis. The average of GPCP and CMAP data is also considered for comparisons, as both data have similar spatial resolution and input measurements. The time periods are chosen in accordance with the availability of data in the public repository. The data sets with different spatial grids allow to diagnose the influence of resolution and period on estimated trends. The study regions are divided in accordance with the climate classifications of IMD, and are termed as Peninsular (PI), West Central (WC), North West (NW), North Central (NC) and North East (NE), as demonstrated in Fig. 2.

## Results and Discussion

### Changes in the rainfall

**Figure 3:**
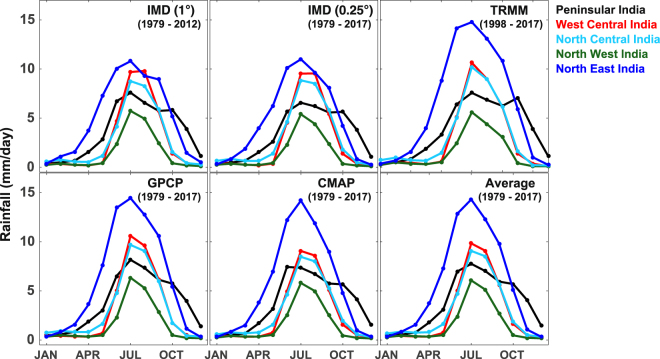
The climatology of monthly averaged rainfall rate (mm/day) over different climate regions of India as observed by rain gauges (IMD Data) and satellite instrument (TRMM), and from the merged rain gauge and satellite data sets (GPCP and CMAP) for the periods mentioned in the figure. Average of GPCP and CMAP data sets for the 1979–2017 period is also shown.

Figure 3 shows the climatology of monthly averaged rainfall rate from various sources such as IMD data at 1° and 0.25° resolutions, TRMM, GPCP, CMAP, and the average of GPCP and CMAP over the respective periods for different climate regions of India. It is to be noted that all data sets provide similar results in all regions, except in the North East, where lower rainfall rates are estimated from the IMD data compared to the satellite and rain gauge measurements. The peak rainfall occurs in the ISMR months, in which maximum is in the North East, about 15 mm/day, and minimum in the North West, about 8 mm/day. However, during the NEMR months, peak rainfall occurs in the Peninsular region, about 6 mm/day, and smallest in the North West, about 1 mm/day. On the other hand, the pre-monsoon rainfall is highest in the North East, about 6 mm/day. The rainfall is very less during the winter season in all regions.

### Seasonal variability: role of local and global climate forcings

**Figure 4:**
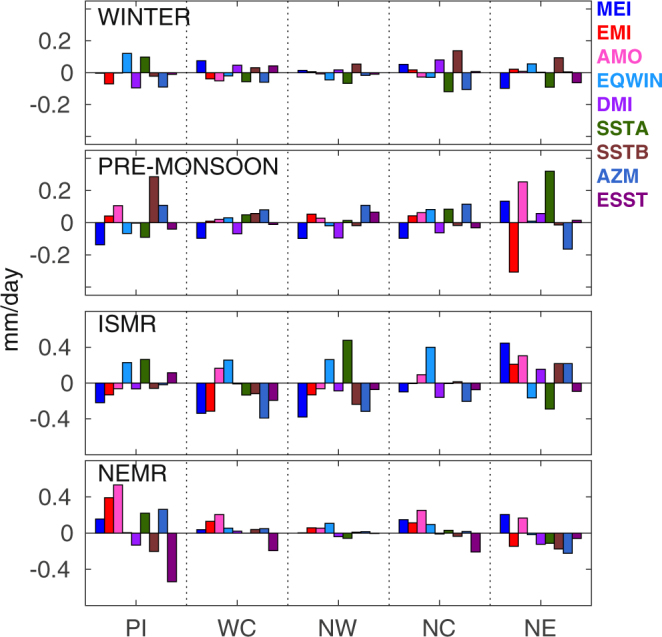
Contribution of different local and remote climate forcings on Indian rainfall in different seasons over 1979–2017 in the Peninsular (PI), West Central (WC), North West (NW), North Central (NC), and North East (NE) India and for winter (January-February), pre-monsoon season (March-April-May), ISMR (June-July-August-September) and NEMR (October-November-December). The North West region takes October-November as the NEMR months and December-January-February as the winter months.

Figure 4 illustrates the variability of proxies (regression coefficient × standard deviation of proxies) contributing to the rainfall for various seasons in different regions, where high values indicate dominating contribution from the respective climate indices. Our analysis reveals that:

(i) the interannual variability of ISMR is mainly driven by the remote forcings such as MEI and EQWIN and the local forcing SSTA in all regions. In addition to these parameters, EMI and AZM also influence the ISMR in the West Central, and SSTB, and AZM affect ISMR in the North West India. In the North East India, ISMR is also controlled by EMI, AMO, SSTB and AZM. (ii) The NEMR variability is mostly controlled by the remote signals of AMO and ESST in most regions. In the Peninsular region, NEMR is also affected by EMI and AZM. In the North East, NEMR is influenced by MEI, EMI, AMO, SSTB and AZM. (iii) The pre-monsoon variability is mostly driven by SSTB in the Peninsular region and by EMI, AMO, and SSTA in the North East. In other regions, rainfall in this season is mainly affected by MEI and AZM index. (iv) The changes in winter rainfall are governed by EQWIN or DMI, and AZM index in the Peninsular India and by the local forcings SSTA and SSTB in the North Central and North East. (v) The effect of local forcings such as the SSTs of Arabian Sea and Bay of Bengal is present in all seasons, but vary with regions. (vi) The AZM influences ISMR while AMO influences NEMR. (vii) The impact of DMI is very less in all seasons while that of EQWIN is strong during ISMR. (viii) The nature of the contribution of climate modes is opposite in the North East as compared to other regions. These results show how the local and remote forcings contribute to the spatial and temporal inhomogeneity of Indian rainfall, and key drivers of the variability in each region and season.

### Drivers of rainfall variability estimated using the MLR method

**Figure 5:**
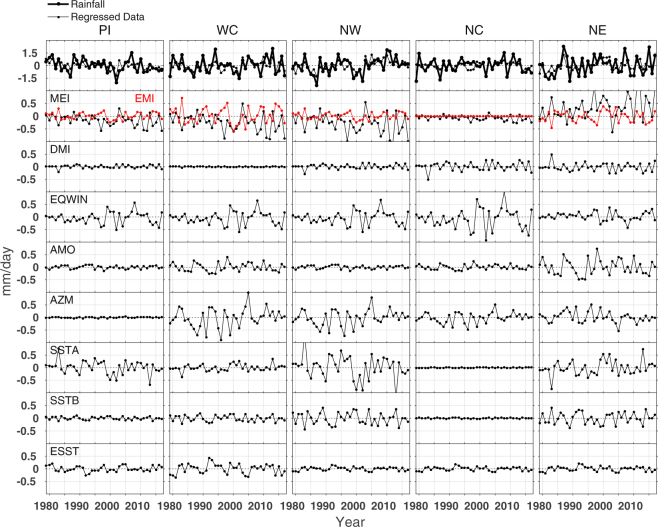
Top panel: The rainfall anomaly computed (thick solid line) for the ISMR (June-July-August-September) from the average of GPCP and CMAP data sets, and the regressed data (thin solid line). Bottom panels (second to ninth from the top): contributions of different climate proxies to the rainfall in each year in the Peninsular (PI), West Central (WC), North West (NW), North Central (NC), and North East (NE) India for the period 1979–2017.

**Figure 6:**
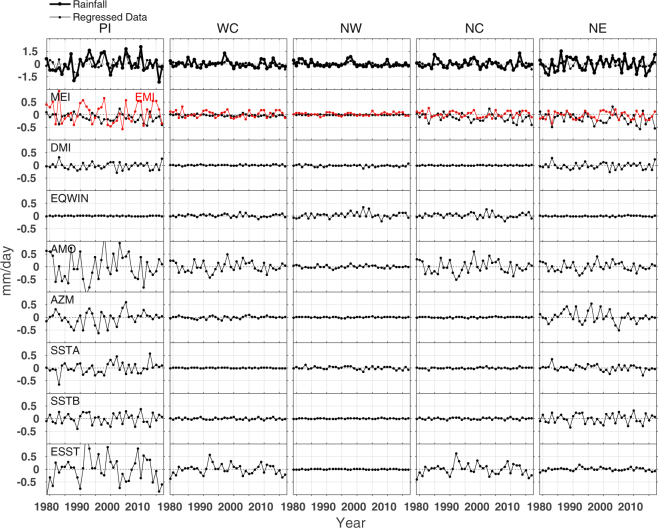
Top panel: The rainfall anomaly computed (thick solid line) for the NEMR (October-November-December) from the average of GPCP and CMAP data sets, and the regressed data (thin solid line). Bottom panels (second to ninth from the top): contributions of different climate proxies to the rainfall in each year in the Peninsular (PI), West Central (WC), North West (NW), North Central (NC), and North East (NE) India for the period 1979–2017. The North West region takes October-November as the NEMR months.

The contributions of different climate processes to the rainfall in different regions as analysed from the average data (average of GPCP and CMAP) for the period 1979–2017 are shown in Figs 5 and 6 for the ISMR and NEMR months, respectively. The top panels represent rainfall anomalies computed (rainfall-climatology) from the observations (thick solid line) and regressed data (thin solid line). The bottom panels (second to ninth) show contributions of climate modes (as noted in the figures) to the rainfall changes. The contributions of the climate forcings to the rainfall from the IMD data at 0.25° × 0.25° latitude × longitude horizontal grids are also analysed for ISMR and NEMR and are shown in the Supplementary Figs S1 and S2, respectively.

#### ISMR

The regressed data clearly replicate the observed rainfall in all regions. The contributions of proxies show large interannual and regional changes. For instance, (i) EP and CP ENSO, EQUINOO and SST of Arabian Sea and Bay of Bengal largely affect the ISMR, about ±0.5 mm/day, in all regions as observed from the respective indices of MEI, EMI, EQWIN, SSTA and SSTB. (ii) The response of EP ENSO is large compared to that of CP ENSO in all regions and the impact of ESST is small compared to other modes. (iii) The ocean component of IOD (DMI) is poorly correlated to the ISMR compared to its atmospheric counterpart (EQWIN). (iv) The peak rainfall in the North East occurs always opposite to that in other regions and all forcings contribute significantly to the rainfall there. In general, during ISMR, winds from the Indian Ocean have two arms when they reach Indian land mass, in which one arm passes through the Arabian Sea to the Western Ghats and the second arm crosses the Bay of Bengal and reaches the Himalayas through a part of North East region. Therefore, the influence of SST of Bay of Bengal is mostly restricted to the Peninsular and North East regions in this season, as demonstrated in the analyses (Fig. 5).

In general, El Niño suppresses and La Niña enhances rainfall and the warm phase of AMO induces rainfall^21^ in India. Similarly, positive IOD phase leads to more rainfall while negative IOD phase leads to less rainfall. Several studies on ISMR^22,23^ state that 1979, 1982, 1985, 1986, 1987, 2002, 2004 and 2009 were drought years and 1983, 1988, 1994 and 2007 were excess rainfall years in India. (v) Among these, 1982, 1987 and 2009 droughts were followed by the positive phase of EP ENSO, and the heavy rainfall in 1988 was due to the occurrence of EP La Niña. (vi) Our anlysis shows that heavy rainfall in 1983 is contributed by the CP La Niña in all regions, but also by AZM in the western regions. (vii) The normal rainfall received in 1997 could be attributed to the positive phase of EQUINOO, although it was a strong El Niño year. (viii) While the extremes in the interannual variation of the ISMR (excess/drought seasons) are explained rather well by ENSO individually or jointly with other climate processes, the spatial variation in the rainfall during these extremes and its links to the climate modes as well as other forcings, such as SST of nearby oceans, were not explored yet, as mentioned previously. The positive IOD year 1994 received heavy rainfall (maximum in the West Central region) and is contributed by AZM. (ix) The year 2002 was dry and is captured in part by the EQUINOO and SSTA forcings in the Peninsular region. In the North West India, the 2002 drought was fully explained by the regressed data through EQUINOO, AZM and SSTA forcings. However, local influences from the Arabian Sea and Bay of Bengal initiate rainfall in the North East and is captured by the regressed data in 2002. (x) The warm phase of IOD in 2007 produced an exceptional rainfall all over India, including North East, as demonstrated by the contributions of EQUINOO in all regions. These suggest that the anomalous and regional differences in rainfall can better be interpreted with multiple climate proxies, and the local forcings play a vital role in regional rainfall differences or spatial inhomogeneity.

#### NEMR

Figure 6 depicts the response of various climate processes to the NEMR. (i) The largest amount of rainfall is received in the Peninsular region and then in the North East region. Therefore, contributions of forcings are also large in the Peninsular India. The North West region receives less amount of rainfall compared to other regions during this season. (ii) The climate modes such as CP ENSO, AMO, and ESST well contribute to the NEMR while contributions of EP ENSO, IOD, and EQUINOO are very small in all regions. (ii) The AZM, SSTA and SSTB contributions are limited to the Peninsular and North East India, with about ±0.5 mm/day. (iv) The contributions of proxies and their region-wise impacts are different for the ISMR and NEMR. In comparison to the ISMR, CP ENSO shows large influence (±1 mm/day) in the Peninsular India, but the IOD and EQUINOO do not affect NEMR in any region. (iv) The AMO and ESST contribute significantly (±0.5 to ±1 mm/day) to the rainfall in all regions for which its influence is strongest in the Peninsular region. The rainfall received in the Peninsular and West Central regions in 1997 was largely contributed by AMO.

#### Pre-monsoon and winter seasons

The rainfall is very limited and sporadic in the pre-monsoon and winter seasons. The analysis was carried out for these too (see Supplementary Figs S3 and S4). The peak rainfall occurs in the Peninsular and North East India and the regressed data capture most features very well during these seasons. In the pre-monsoon season, contributions of CP ENSO, AMO, AZM, and SSTA are large in the North East in agreement with the heavy rainfall there, but SSTB contributes to much of the variability in the Peninsular region. In winter, rainfall is very small and is mainly dominated by AZM and the local forcing SSTA in most regions. The largest rainfall occurs in the Peninsular India compared to other regions and is also contributed by EQWIN and SSTA. In brief, the local forcings dominate the irregular and small amount of rainfall during the pre-monsoon and winter seasons.

### Trends in Indian rainfall

#### MLR method

The long-term trends in rainfall are calculated using the MLR method and results with twice the standard deviation (95% confidence level) are shown in Table 1. In general, all data sets show similar trends during the 1979–2017 period in all seasons, indicating the consistency of measurements from different instruments. The trends are also estimated from the average of GPCP and CMAP data for the period 1998–2017 to compare with those from TRMM and the results are very similar. The trends are largest (in absolute value) for the ISMR and smallest for winter. Although most estimates are not statistically significant at the 95% confidence level, the NEMR trends estimated from the TRMM data show a significant negative trend (−1.10 ± 1.00 mm/day/dec) in the Peninsular region. All other data sets show significant positive trends (0.43 ± 0.30 mm/day/dec) for the ISMR in the North West India. The GPCP data show a significant positive trend (0.26 ± 0.25 mm/day/dec) for the pre-monsoon season in the North East India.

**Table 1:** The trends (mm/day/dec) estimated using the multiple linear regression method from the rainfall anomalies computed for the periods 1979–2012, 1979–2017 and 1998–2017, as available from each instrument and the average (GPCP and CMAP) data for winter, pre-monsoon season, ISMR and NEMR. Trends are shown with 95% confidence level for five different climate regions of India. The statistically significant values are shown in bold. The average of GPCP and CMAP data are analysed for both 1979–2017 and 1998–2017 periods and these periods are also shown in bold.

Dataset	Year	Winter	Pre-monsoon	ISMR	NEMR
**Peninsular India**					
IMD (1° × 1°)	1979–2012	−0.06 ± 0.16	0.13 ± 0.24	0.34 ± 0.64	0.32 ± 0.42
IMD (0.25° × 0.25°)	1979–2017	0.01 ± 0.15	0.16 ± 0.21	0.19 ± 0.32	0.16 ± 0.28
GPCP (2.5° × 2.5°)	1979–2017	−0.03 ± 0.15	**0.19** ± **0.18**	−0.10 ± 0.25	0.00 ± 0.29
CMAP (2.5° × 2.5°)	1979–2017	−0.08 ± 0.16	0.16 ± 0.20	−0.07 ± 0.30	0.06 ± 0.32
Average (2.5° × 2.5°)	**1979**–**2017**	−0.05 ± 0.15	0.18 ± 0.19	−0.08 ± 0.26	0.03 ± 0.30
Average (2.5° × 2.5°)	**1998**–**2017**	−0.21 ± 0.28	0.07 ± 0.48	−0.01 ± 0.97	−0.77 ± 0.86
TRMM (0.25° × 0.25°)	1998–2017	−0.20 ± 0.29	0.16 ± 0.50	0.23 ± 1.08	−**1.10** ± **1.00**
**West Central India**					
IMD (1° × 1°)	1979–2012	−0.10 ± 0.12	0.00 ± 0.07	−0.11 ± 0.43	0.02 ± 0.18
IMD (0.25° × 0.25°)	1979–2017	−0.00 ± 0.13	0.03 ± 0.06	−0.02 ± 0.38	−0.06 ± 0.14
GPCP (2.5° × 2.5°)	1979–2017	−0.04 ± 0.10	0.05 ± 0.08	0.07 ± 0.34	−0.03 ± 0.14
CMAP (2.5° × 2.5°)	1979–2017	−0.04 ± 0.11	0.02 ± 0.07	0.06 ± 0.31	−0.05 ± 0.15
Average (2.5° × 2.5°)	**1979**–**2017**	−0.04 ± 0.11	0.03 ± 0.07	0.07 ± 0.31	−0.04 ± 0.15
Average (2.5° × 2.5°)	**1998**–**2017**	−0.04 ± 0.34	0.12 ± 0.29	0.20 ± 0.73	−0.16 ± 0.33
TRMM (0.25° × 0.25°)	1998–2017	−0.02 ± 0.41	0.10 ± 0.29	0.12 ± 0.88	−0.20 ± 0.36
**North West India**					
IMD (1° × 1°)	1979–2012	−0.03 ± 0.07	−0.06 ± 0.07	0.35 ± 0.40	−0.11 ± 0.18
IMD (0.25° × 0.25°)	1979–2017	−0.01 ± 0.06	−0.02 ± 0.08	0.33 ± 0.34	−0.09 ± 0.12
GPCP (2.5° × 2.5°)	1979–2017	0.00 ± 0.07	−0.00 ± 0.08	**0.43** ± **0.30**	−0.06 ± 0.11
CMAP (2.5° × 2.5°)	1979–2017	−0.02 ± 0.06	−0.07 ± 0.11	**0.36** ± **0.29**	−0.09 ± 0.14
Average (2.5° × 2.5°)	**1979**–**2017**	−0.01 ± 0.06	−0.04 ± 0.10	**0.40** ± **0.29**	−0.07 ± 0.12
Average (2.5° × 2.5°)	**1998**–**2017**	0.00 ± 0.25	0.15 ± 0.20	**0.80** ± **0.69**	−0.01 ± 0.28
TRMM (0.25° × 0.25°)	1998–2017	−0.05 ± 0.29	0.18 ± 0.20	0.83 ± 0.87	−0.06 ± 0.30
**North Central India**					
IMD (1° × 1°)	1979–2012	−0.07 ± 0.15	−0.06 ± 0.11	−0.15 ± 0.39	0.04 ± 0.17
IMD (0.25° × 0.25°)	1979–2017	0.06 ± 0.14	−0.10 ± 0.11	−0.11 ± 0.33	0.03 ± 0.19
GPCP (2.5° × 2.5°)	1979–2017	−0.05 ± 0.13	−0.01 ± 0.11	−0.07 ± 0.32	−0.02 ± 0.16
CMAP (2.5° × 2.5°)	1979–2017	−0.05 ± 0.11	−0.06 ± 0.12	0.06 ± 0.29	0.06 ± 0.20
Average (2.5° × 2.5°)	**1979**–**2017**	−0.05 ± 0.12	−0.04 ± 0.11	−0.00 ± 0.29	0.02 ± 0.18
Average (2.5° × 2.5°)	**1998**–**2017**	−0.01 ± 0.48	0.07 ± 0.36	−0.19 ± 0.83	−0.13 ± 0.42
TRMM (0.25° × 0.25°)	1998–2017	0.02 ± 0.68	0.10 ± 0.37	0.06 ± 1.12	−0.22 ± 0.33
**North East India**					
IMD (1° × 1°)	1979–2012	−0.13 ± 0.17	−0.51 ± 0.66	−0.59 ± 0.81	0.20 ± 0.52
IMD (0.25° × 0.25°)	1979–2017	−0.07 ± 0.17	−0.11 ± 0.37	−0.28 ± 0.46	0.10 ± 0.26
GPCP (2.5° × 2.5°)	1979–2017	−0.06 ± 0.12	**0.26** ± **0.25**	−0.01 ± 0.36	0.12 ± 0.29
CMAP (2.5° × 2.5°)	1979–2017	−0.08 ± 0.14	0.12 ± 0.29	0.04 ± 0.55	0.15 ± 0.28
Average (2.5° × 2.5°)	**1979**–**2017**	−0.07 ± 0.12	0.19 ± 0.23	0.02 ± 0.39	0.14 ± 0.27
Average (2.5° × 2.5°)	**1998**–**2017**	−0.08 ± 0.43	0.46 ± 0.62	0.19 ± 1.02	0.01 ± 0.72
TRMM (0.25° × 0.25°)	1998–2017	−0.11 ± 0.54	0.38 ± 0.81	0.62 ± 1.36	0.19 ± 0.90

The trend analysis with all four data sets in 1979–2017 shows that the ISMR is decreasing and NEMR is increasing in the Peninsular, North Central and North East regions, whereas opposite trends are observed in the West Central and North West India. The pre-monsoon rainfall is increasing in the Peninsular, West Central and North East India and decreasing in other regions while the winter rainfall is decreasing in all regions. None of these trends mentioned are significant at 95% confidence level.

The results are consistent with the analyses of Kumar *et al*.^24^, who also showed an insignificant negative trend in the ISMR (−0.43 mm/yr), positive trends in the NEMR (0.11 mm/yr) and pre-monsoon season (0.04 mm/yr) over different regions of India in the 1871–2005 period. They also showed insignificant negative trends in rainfall (−0.01 mm/yr) in the North East and North West India and positive trends (0.01 to 0.05 mm/yr) in other regions during winter. Similarly, Das *et al*.^25^ found insignificant negative trend (−0.62 mm/yr) in the ISMR over North East in 1961–2010, as estimated in our study for the 1979–2017 period. However, they estimated significant positive trends for other seasons. Our study also shows significant positive trends for pre-monsoon season, but insignificant positive trends for NEMR. The slight differences in the computed trends among different studies can be due to the differences in the periods of estimates.

#### Simple linear regression method

The trends are also estimated using the simple linear regression method (without including the climate modes in the model) and are shown in Table 2. It shows that ISMR is decreasing in the Peninsular, North Central and North East regions but increasing in all other regions, same as that obtained from the MLR method. However, statistically significant positive trends are estimated for North West (0.82 mm/day/dec) during the 1998–2017 period and North East (0.53 mm/day/dec) during the 1979–2017 period. On the other hand, NEMR is increasing in the Peninsular and North East India while decreasing in other regions, although these are not statistically significant. A similar result is estimated from the MLR method except for the North Central India. The pre-monsoon rainfall shows a statistically significant positive trend (0.18 mm/day/dec) in the Peninsular, significant negative trend (−0.69 mm/day/dec) in the North East and insignificant negative trends in the West Central, North West and North Central regions in 1979–2017. The winter rainfall shows insignificant negative trends in all regions while a significant negative trend (−0.24 mm/day/dec) is estimated in the Peninsular region during the 1998–2017 period.

**Table 2:** The trends (mm/day/dec) estimated using simple linear regression method (without including climate modes in the model) from the rainfall anomalies for the periods 1979–2012, 1979–2017 and 1998–2017, as available from each instrument and the average (GPCP and CMAP) data for winter, pre−monsoon season, ISMR and NEMR. Trends are shown with 95% confidence level for five different climate regions of India. The statistically significant values are shown in bold. The average of GPCP and CMAP data are analysed for both 1979–2017 and 1998–2017 periods and these periods are also shown in bold.

Dataset	Year	Winter	Pre-monsoon	ISMR	NEMR
**Peninsular India**					
IMD (1° × 1°)	1979–2012	−0.05 ± 0.14	0.16 ± 0.22	0.47 ± 0.48	0.25 ± 0.36
IMD (0.25° × 0.25°)	1979–2017	0.01 ± 0.10	**0.15** ± **0.13**	0.01 ± 0.21	0.04 ± 0.23
GPCP (2.5° × 2.5°)	1979–2017	−0.05 ± 0.12	**0.18** ± **0.16**	−0.21 ± 0.21	0.04 ± 0.27
CMAP (2.5° × 2.5°)	1979–2017	−0.08 ± 0.12	0.14 ± 0.17	−0.18 ± 0.24	0.11 ± 0.29
Average (2.5° × 2.5°)	**1979**–**2017**	−0.07 ± 0.12	0.16 ± 0.16	−0.20 ± 0.21	0.07 ± 0.28
Average (2.5° × 2.5°)	**1998**–**2017**	−**0.24** ± **0.20**	−0.04 ± 0.39	−0.06 ± 0.61	−0.44 ± 0.76
TRMM (0.25° × 0.25°)	1998–2017	−0.22 ± 0.22	0.04 ± 0.38	0.11 ± 0.71	−0.71 ± 0.93
**West Central India**					
IMD (1° × 1°)	1979–2012	−0.10 ± 0.09	0.01 ± 0.06	0.04 ± 0.37	−0.06 ± 0.15
IMD (0.25° × 0.25°)	1979–2017	0.07 ± 0.09	−0.01 ± 0.07	0.31 ± 0.30	−0.02 ± 0.13
GPCP (2.5° × 2.5°)	1979–2017	−0.04 ± 0.08	0.06 ± 0.07	0.04 ± 0.28	−0.05 ± 0.12
CMAP (2.5° × 2.5°)	1979–2017	−0.05 ± 0.09	0.03 ± 0.07	0.09 ± 0.27	−0.06 ± 0.12
Average (2.5° × 2.5°)	**1979**–**2017**	−0.05 ± 0.08	0.05 ± 0.07	0.06 ± 0.27	−0.05 ± 0.12
Average (2.5° × 2.5°)	**1998**–**2017**	0.02 ± 0.21	0.03 ± 0.20	0.23 ± 0.77	−0.07 ± 0.32
TRMM (0.25° × 0.25°)	1998–2017	0.04 ± 0.25	0.04 ± 0.21	0.20 ± 0.91	−0.09 ± 0.34
**North West India**					
IMD (1° × 1°)	1979–2012	−0.01 ± 0.05	−0.04 ± 0.08	0.31 ± 0.36	−0.09 ± 0.12
IMD (0.25° × 0.25°)	1979–2017	0.02 ± 0.04	−0.03 ± 0.05	0.04 ± 0.29	−0.07 ± 0.10
GPCP (2.5° × 2.5°)	1979–2017	0.00 ± 0.05	0.02 ± 0.07	0.27 ± 0.27	−0.06 ± 0.09
CMAP (2.5° × 2.5°)	1979–2017	−0.01 ± 0.04	−0.04 ± 0.09	0.21 ± 0.27	−0.08 ± 0.10
Average (2.5° × 2.5°)	**1979**–**2017**	−0.00 ± 0.04	−0.01 ± 0.08	0.24 ± 0.26	−0.07 ± 0.09
Average (2.5° × 2.5°)	**1998**–**2017**	0.02 ± 0.14	0.11 ± 0.14	**0.76** ± **0.67**	−0.12 ± 0.24
TRMM (0.25° × 0.25°)	1998–2017	0.00 ± 0.17	0.14 ± 0.17	**0.82** ± **0.81**	−0.22 ± 0.28
**North Central India**					
IMD (1° × 1°)	1979–2012	−0.09 ± 0.12	−0.04 ± 0.12	0.05 ± 0.30	−0.06 ± 0.15
IMD (0.25° × 0.25°)	1979–2017	0.05 ± 0.11	−0.03 ± 0.10	0.08 ± 0.30	−0.06 ± 0.15
GPCP (2.5° × 2.5°)	1979–2017	−0.06 ± 0.10	0.03 ± 0.09	−0.08 ± 0.25	−0.04 ± 0.12
CMAP (2.5° × 2.5°)	1979–2017	−0.07 ± 0.09	−0.01 ± 0.10	0.13 ± 0.24	0.00 ± 0.15
Average (2.5° × 2.5°)	**1979**–**2017**	−0.06 ± 0.10	0.01 ± 0.09	0.03 ± 0.23	−0.02 ± 0.14
Average (2.5° × 2.5°)	**1998**–**2017**	0.04 ± 0.31	0.06 ± 0.21	−0.23 ± 0.63	−0.09 ± 0.40
TRMM (0.25° × 0.25°)	1998–2017	0.11 ± 0.39	0.10 ± 0.24	−0.16 ± 0.88	−0.18 ± 0.33
**North East India**					
IMD (1° × 1°)	1979–2012	−0.17 ± 0.18	−**0.69** ± **0.59**	−0.51 ± 0.66	−0.02 ± 0.43
IMD (0.25° × 0.25°)	1979–2017	0.01 ± 0.10	0.02 ± 0.23	**0.53** ± **0.19**	0.07 ± 0.17
GPCP (2.5° × 2.5°)	1979–2017	−0.08 ± 0.08	0.13 ± 0.27	−0.02 ± 0.30	0.02 ± 0.24
CMAP (2.5° × 2.5°)	1979–2017	−0.10 ± 0.11	0.08 ± 0.28	0.37 ± 0.45	0.05 ± 0.24
Average (2.5° × 2.5°)	**1979**–**2017**	−0.09 ± 0.09	0.10 ± 0.25	0.17 ± 0.32	0.04 ± 0.23
Average (2.5° × 2.5°)	**1998**–**2017**	−0.09 ± 0.25	−0.17 ± 0.66	−0.17 ± 0.84	−0.22 ± 0.60
TRMM (0.25° × 0.25°)	1998–2017	−0.12 ± 0.30	−0.23 ± 0.77	−0.05 ± 1.06	−0.09 ± 0.78

## Conclusions

This study introduces multiple linear regression technique for the evaluation of the contribution of different local and remote drivers of climate change and to estimate trends in rainfall over different regions across four seasons in India. The importance of this method is that it uses a time-variant parameter for determining trends in rainfall and provides quantitative evaluation of trends in rainfall. Also, trends in climate modes are removed before applying in the regression model and hence, the computed trends would be apparently free from the trends of proxies used in the regression model. This study assesses combined relationship between Indian rainfall and different local and global climate processes.

Our study finds that (i) changes in the Arabian Sea and Bay of Bengal surface temperatures, equatorial zonal winds, Atlantic zonal mode, EP ENSO and CP ENSO control the variability of ISMR in all regions. (ii) The CP ENSO and the changes in SST of North Atlantic and extratropical oceans decide the variability of NEMR. (iii) The pre-monsoon rainfall variability is controlled by CP ENSO, AMO and SSTA, whereas the winter rainfall variability is dominated by EQWIN or DMI, AZM and the local forcings SSTA and SSTB. (iv) The Bay of Bengal surface temperature is the major driver of rainfall variability in the North East in all seasons. (v) These results unravel the role of a number of climate modes including the local forcings, in addition to the commonly used global climate forcings, in determining the variability of rainfall distribution over India. (vi) An insignificant negative trend is estimated for ISMR and positive trend for NEMR in the Peninsular, North Central and North East regions, and the trends are opposite in other regions. Statistically significant positive trend (0.43 mm/day/dec) at 95% confidence level is found in the North West India for ISMR, as estimated from the IMD, GPCP and CMAP data.

We have introduced a multiple linear regression model for the variability and trend analyses of rainfall, and the model with nine detrended and lagged climate parameters fits well with the observed rainfall in all seasons. Since similar statistical techniques are used for predicting weather and extreme weather events, the method presented in this study can be applied to improve the existing prediction models or weather forecasting models of Indian monsoon. Henceforth, our study unveils some interesting facts on the variability of Indian rainfall, both in terms of contributing factors and long-term trends. The observed changes in rainfall also have signatures of the impact of regional and global climate change, and hence, corroborate the importance of continued monitoring and long-term analyses of rainfall, as these kinds of studies have great significance in the context of climate impact assessments and government level decisions.

## Methods

The gridded rainfall data compiled from rain gauges deployed at different places in India by India Meteorological Department (IMD) are used. The IMD data are available on 1° × 1° and 0.25° × 0.25° latitude × longitude horizontal resolution for the periods 1979–2012 and 1979–2017, respectively. In addition, the precipitation data are taken from the combined rain gauge and satellite observations such as Global Precipitation Climatology Project (GPCP) version 2.3 and Climate Prediction Center (CPC) Merged Analysis of Precipitation (CMAP), and are available on 2.5° × 2.5° spatial resolution for the period 1979–2017. The average of GPCP and CMAP data is also considered for the analysis. The rainfall data from the satellite instrument, Tropical Rainfall Measuring Mission (TRMM) version 3B43v7, with a horizontal resolution of 0.25° × 0.25° from 1998 to 2017 are also used.

The major drivers representing different climate processes, which can significantly affect regional and global rainfall pattern such as eastern Pacific (EP) El Niño Southern Oscillation (ENSO), central Pacific (CP) ENSO, Atlantic Multidecadal Oscillation (AMO), Indian Ocean Dipole (IOD), Equatorial Indian Ocean Oscillation (EQUINOO), Atlantic Zonal Mode (AZM), and Extratropical Sea Surface temperature (ESST) are used. The Multivariate ENSO Index (MEI) based on six atmospheric and oceanic variables in the Niño 3.4 region (5°N −5°S and 170°W − 120°W) is used to characterise the intensity of EP ENSO. The El Niño Modoki Index (EMI) derived from the difference in area averaged SST anomalies in the regions of 10°S − 10°N and 165°E − 140°W; 15°S − 5°N and 110°W − 70°W; and 10°S − 20°N and 125°E − 145°E using the method provided in^7^, is considered as the CP ENSO index^26^. The annual average of SST anomalies at 0° − 60°N and 75°W − 7.5°W is taken as the AMO index for studying the impact of SST variability in the North Atlantic Ocean on Indian rainfall. The index corresponding to the AZM is computed from the area averaged SST anomaly over 3°S − 3°N and 20°W − 0°W. The Dipole Mode Index (DMI), the SST gradient between the western equatorial Indian Ocean (10°S − 10°N and 50°E − 70°E) and the south eastern equatorial Indian Ocean (10°S − 0°N and 90°E − 110°E) is considered as the ocean component of IOD. The EQUINOO is the atmospheric counterpart of IOD, and is based on the surface zonal wind anomaly over the central equatorial Indian Ocean (2.5°S − 2.5°N and 60°E − 90°E). The negative of this wind anomaly, normalized by its standard deviation is used as the index of EQUINOO, termed as Equatorial zonal Wind Index (EQWIN)^27^. However, the positive IOD events are associated with EQUINOO, but is not true for negative IODs. In fact, IOD and EQUINOO are loosely coupled. Therefore, the correlation between EQWIN and ISMR is better than that between DMI and ISMR^28,29^ although these two indices represent IOD. The extratropical SST is considered as the area-averaged SST anomaly of the extratropical oceanic region of 15°N − 75°N and 100°E − 5°W.

Apart from these, two new proxies are constructed to study the influences of SSTs of Arabian Sea and Bay of Bengal on Indian rainfall. The large SST variability region is determined from the spatial variance computed for the JJAS months in 1979–2017, and the regions considered are 10°N − 15°N and 51°E − 60°E for Arabian Sea and 6°N − 10°N and 78°E − 82°E for Bay of Bengal. Then, corresponding indices are computed for each month (January to December) using area-averaged SST for those regions. The monthly Extended Reconstructed SST (ERSST) version 5 data^30^ are used for making these indices. All these climate modes are detrended, and are normalized to unity to be used in the regression model. The lagged combination of these parameters is used in the model and the considered lag is zero month for CP ENSO and one month for other proxies.

The analysis is carried out using monthly mean rainfall anomaly (mm/day) averaged for the winter season (January–February), pre-monsoon season (March–April–May), ISMR (June–July–August–September) and NEMR (October–November–December), as defined by IMD. The North West region takes October–November as the NEMR months and December–January–February as winter. Also, region-wise analysis is performed to investigate the spatial inhomogeneity of rainfall in relation to the climate modes.

The MLR model used to fit the rainfall data in terms of explanatory variables is:

1 $$\begin{array}{rcl}Y(z) & = & {C}_{A}A(z)+{C}_{z}z+{C}_{MEI}MEI(z)+{C}_{EMI}EMI(z)+{C}_{AMO}AMO(z)\\  &  & +{C}_{AZM}AZM(z)+{C}_{DMI}DMI(z)+{C}_{EQWIN}EQWIN(z)\\  &  & +{C}_{ESST}ESST(z)+{C}_{SSTA}SSTA(z)+{C}_{SSTB}SSTB(z)+\varepsilon (z)\end{array}$$

where Y is the rainfall data, z is years, A is a constant level term (taken as value 1), C _*z*_ is linear trend, C_*A*_, C_*MEI*_, C_*EMI*_, C_*AMO*_, C_*AZM*_, C_*DMI*_, C_*EQWIN*_, C_*ESST*_, C_*SSTA*_ and C_*SSTB*_ are the regression coefficients of the time series of constant term, EP ENSO, CP ENSO, AMO, AZM, DMI, EQWIN, ESST, SSTA, and SSTB, respectively, that determines the contribution of each variable given the other variables are held constant and *ε* is the residual. This model can be expressed in vector form as

2 $$\vec{Y}=\vec{C}\vec{X}+\vec{{\varepsilon }}$$

where X (z × p) is the proxy data and C (p × 1) holds the regression coefficient of each proxy. p is the number of proxy terms including the constant used in the regression model and is eleven in this study. The standard error of the regression coefficient is calculated by minimising the model using the generalised least squares method^31^ by setting

3 $$\hat{C}={({\vec{X}}^{T}\vec{X})}^{-1}{\vec{X}}^{T}\vec{Y}$$

The residual is computed as

4 $$\hat{{\rm{\varepsilon }}}=\vec{Y}-\hat{C}\vec{X}$$

The error standard deviation is

5 $$\hat{{\rm{\sigma }}}=\sqrt{\frac{\displaystyle \sum _{z}{[Y(z)-CX(z)]}^{2}}{z-p}}$$

The variances of the regression coefficients are the diagonal elements of the standard error matrix

6 $${\sigma }_{p}^{2}={\hat{\sigma }}^{2}{({\vec{X}}^{T}\vec{X})}^{-1}$$

The standard deviation of the regression coefficient is dependent on the variance and autocorrelation (*ϕ*) of the residual. Therefore, autocorrelation is also incorporated in the estimation of standard deviation without which a precise trend estimate is not possible and would falsely estimate the 95% confidence level.

7 $${{\sigma }}_{p}={\hat{{\rm{\sigma }}}}_{p}\times \sqrt{\frac{1+{\varphi }}{1-{\varphi }}}$$

The trend is said to be statistically significant if it is greater than twice its standard deviation (approximately 95% confidence level) in this study.

There are certain assumptions in using MLR model that (i) the explanatory parameters are not highly correlated each other. (ii) The residuals are normally distributed. (iii) The error term is not auto-correlated as it affects the error estimation. Therefore, the presented model is made by verifying all these factors. The multicollinearity among the climate indices is checked using the tolerance and variance inflation factor^32^ and found that model is free from multicollinearity among the variables. Also, existence of autocorrelation of residuals is evaluated using Durbin-Watson test and is inside the limits as stated in^33^. Therefore, the considered MLR model satisfies the conditions of goodness of fit of the models.

A study by Krishnaswamy *et al*.^34^ reported that there exists nonstationarity and nonlinearity among the climate processes, especially between ENSO and IOD. This nonlinear relationship among the climate modes can also be studied using multiple non-linear regression model as done by Esha and Imteaz^35^.

## Electronic supplementary material

Supplementary Data
