# Supplementary material for: The local and global climate forcings induced inhomogeneity of Indian rainfall
Source: Sci Rep. 2018 Apr 16;8:6026. doi: 10.1038/s41598-018-24021-x (PMC5902448; doi:10.1038/s41598-018-24021-x)
Supplement: Supplementary file 1 — Supplementary Data [file 41598_2018_24021_MOESM1_ESM.pdf]

Supplementary Material

THE LOCAL AND GLOBAL CLIMATE FORCINGS INDUCED INHOMOGENEITY OF INDIAN RAINFALL

P. J. NAIR[1,*], A. CHAKRABORTY[1], H. VARIKODEN[2], P. A. FRANCIS[3], AND J. KUTTIPPURATH[1]

[1] CORAL, Indian Institute of Technology Kharagpur, West Bengal, India
[2] Indian Institute of Tropical Meteorology, Pashan, Pune-411008, India
[3] ESSO-Indian National Centre for Ocean Information Services, Hyderabad, India
[*] pjnnair@yahoo.in

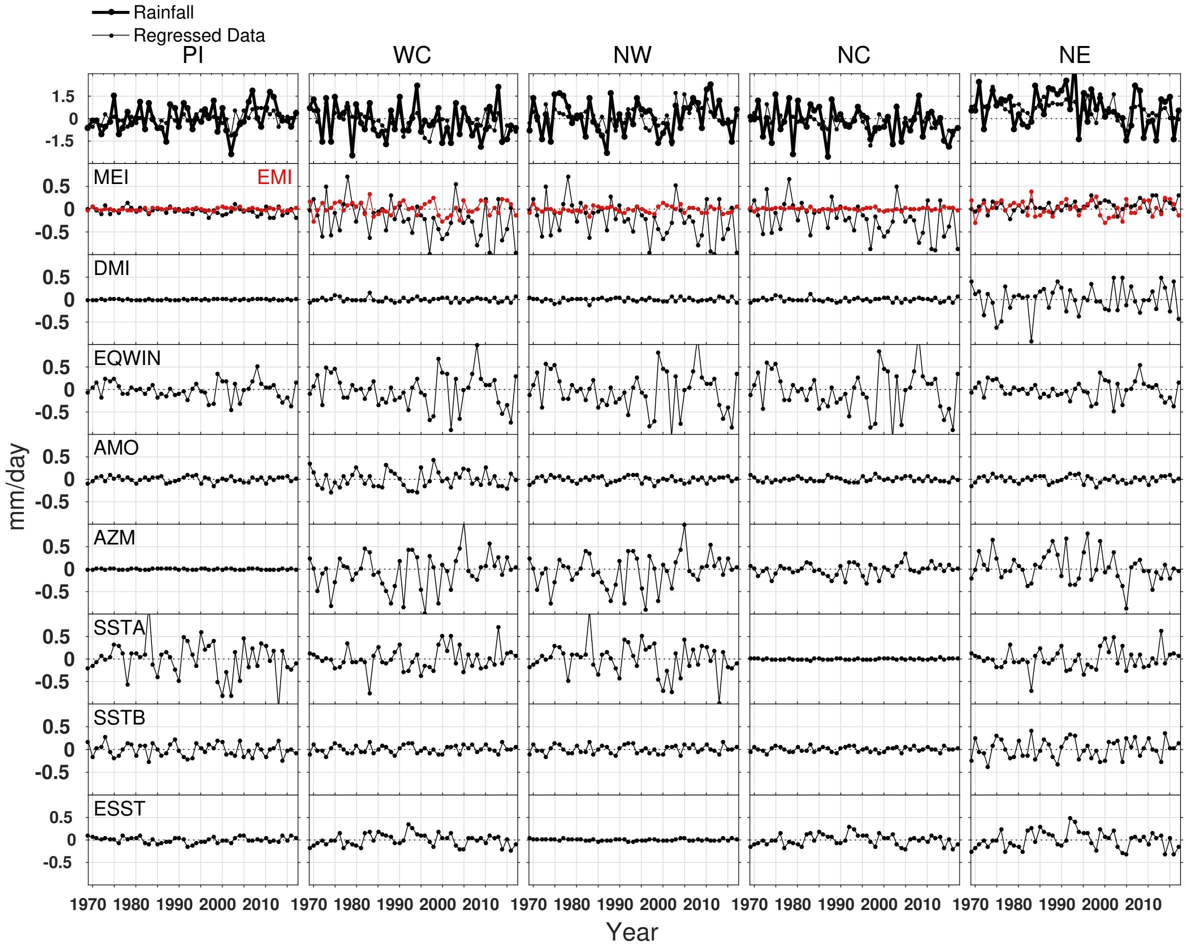

FIG. S 1. Top panel: The rainfall anomaly computed (thick solid line) for the ISMR (June-July-August-September) from the IMD data at 0.25°× 0.25° resolution, and the regressed data (thin solid line). Bottom panels (second to ninth from the top): contributions of different climate proxies to the rainfall in each year in the Peninsular (PI), West Central (WC), North West (NW), North Central (NC), and North East (NE) India for the period 1979–2017.

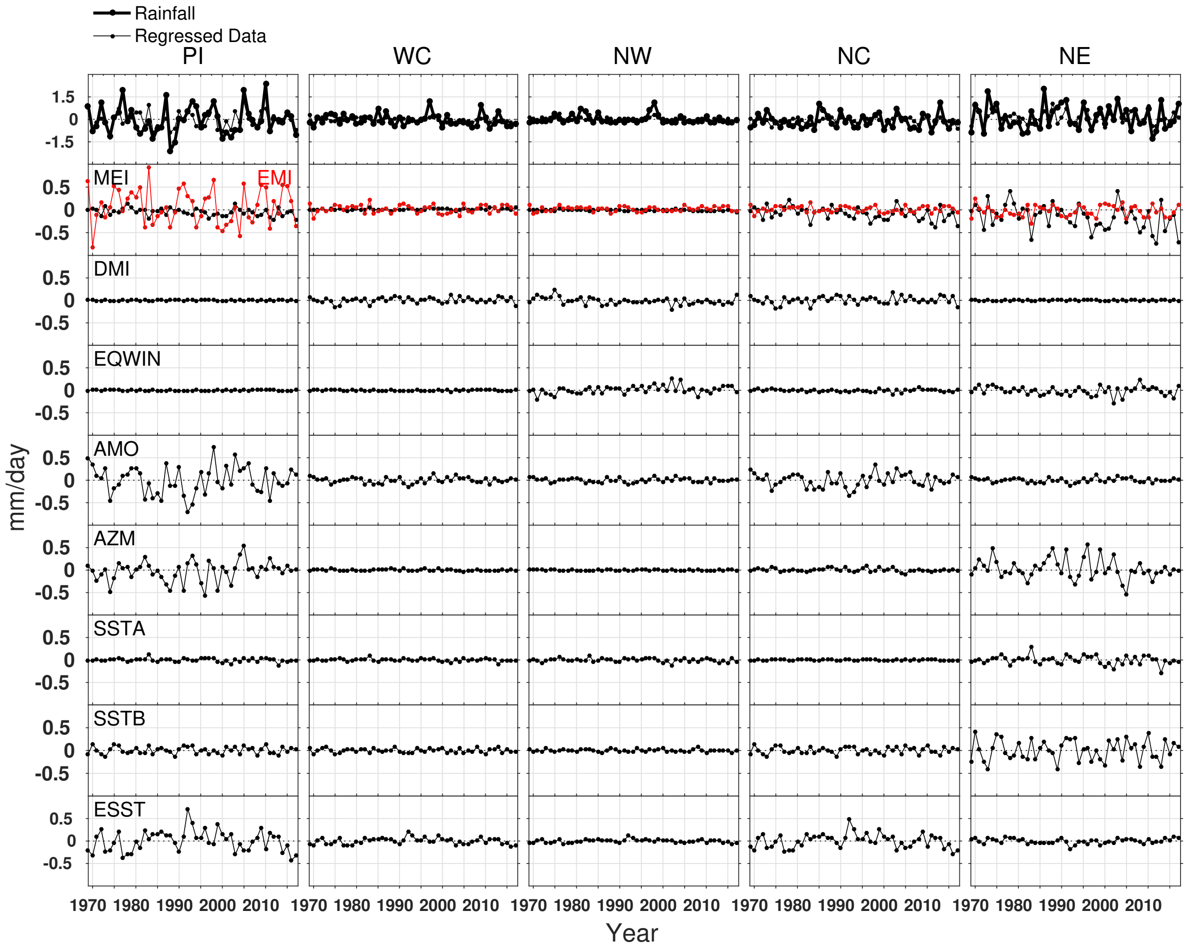

Fig. S 2. Top panel: The rainfall anomaly computed (thick solid line) for the NEMR (October-November-December) from the IMD data at 0.25°× 0.25° resolution, and the regressed data (thin solid line). Bottom panels (second to ninth from the top): contributions of different climate proxies to the rainfall in each year in the Peninsular (PI), West Central (WC), North West (NW), North Central (NC), and North East (NE) India for the period 1979–2017. The North West region takes October-November as the NEMR months.

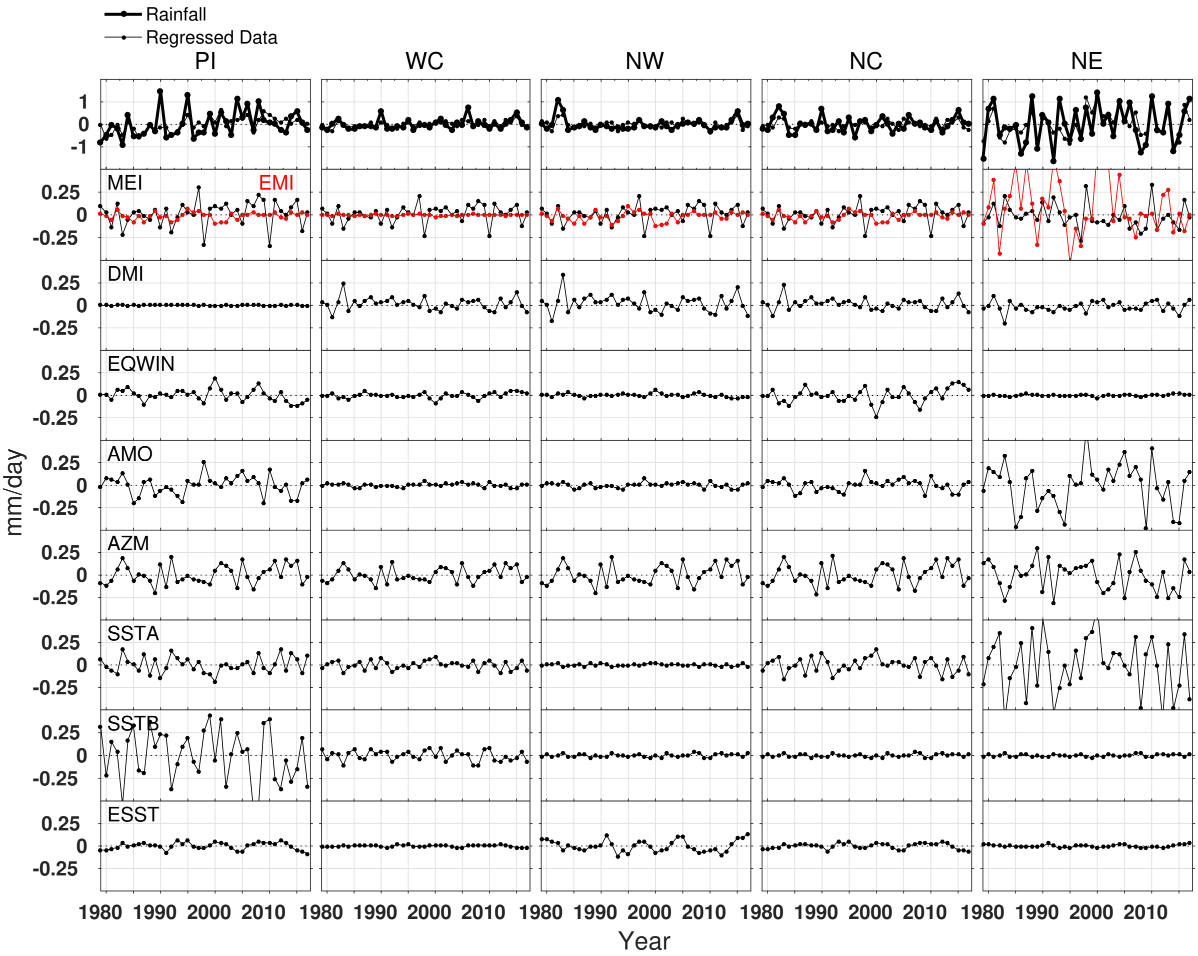

Fig. S 3. Top panel: The rainfall anomaly computed (thick solid line) for the pre-monsoon (March-April-May) season from the average of GPCP and CMAP data sets, and the regressed data (thin solid line). Bottom panels (second to ninth from the top): contributions of different climate proxies to the rainfall in each year in the Peninsular (PI), West Central (WC), North West (NW), North Central (NC), and North East (NE) India for the period 1979–2017.

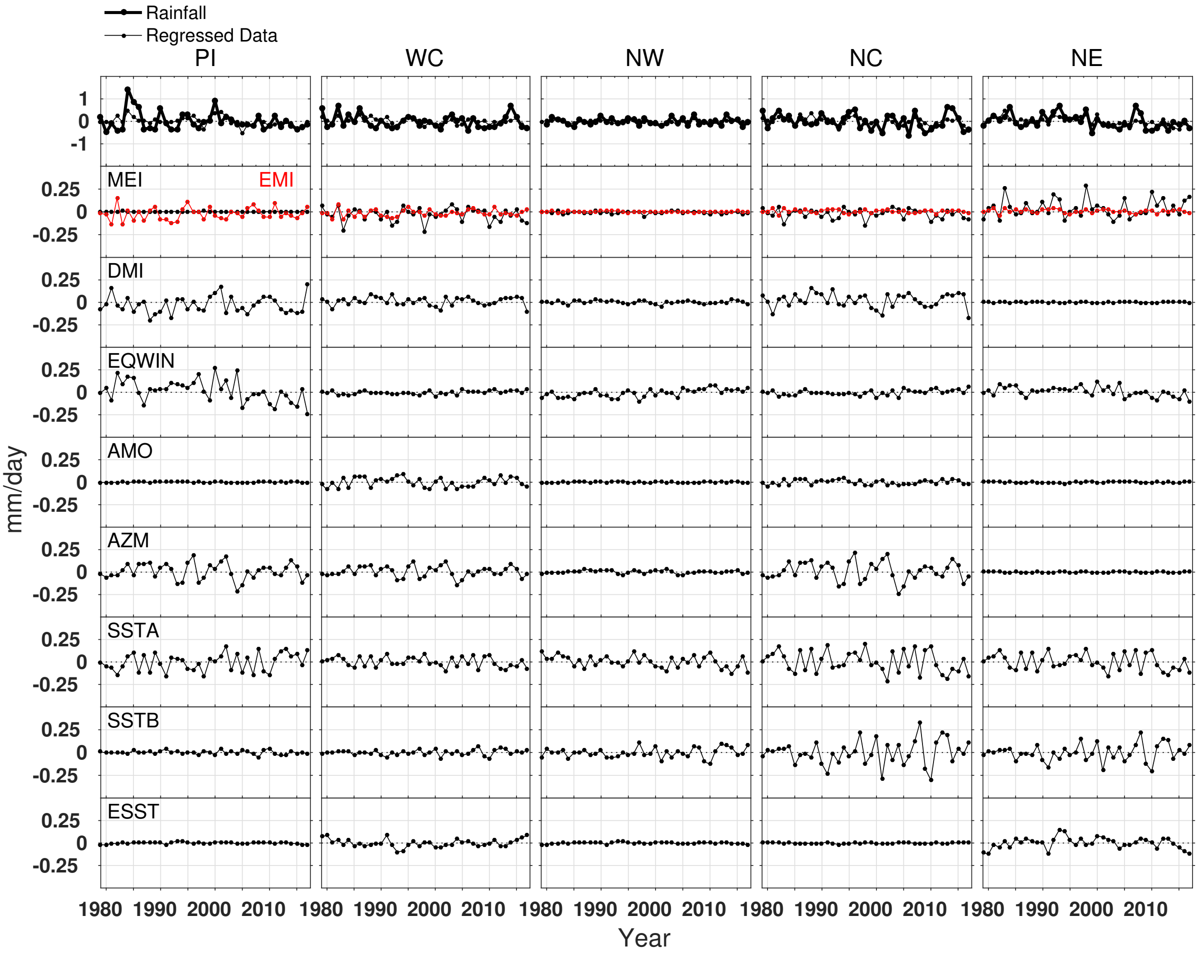

Fig. S 4. Top panel: The rainfall anomaly computed (thick solid line) for the winter (January-February) season from the average of GPCP and CMAP data sets, and the regressed data (thin solid line). Bottom panels (second to ninth from the top): contributions of different climate proxies to the rainfall in each year in the Peninsular (PI), West Central (WC), North West (NW), North Central (NC), and North East (NE) India for the period 1979–2017. The North West region takes December-January-February as the winter months.
